# Algae in Fish Feed: Performances and Fatty Acid Metabolism in Juvenile Atlantic Salmon

**DOI:** 10.1371/journal.pone.0124042

**Published:** 2015-04-15

**Authors:** Fernando Norambuena, Karen Hermon, Vanessa Skrzypczyk, James A. Emery, Yoni Sharon, Alastair Beard, Giovanni M. Turchini

**Affiliations:** 1 School of Life and Environmental Sciences, Deakin University, Warrnambool, Victoria, Australia; 2 MBD Energy ltd. Melbroune, Victoria, Australia; Scottish Association for Marine Science, UNITED KINGDOM

## Abstract

Algae are at the base of the aquatic food chain, producing the food resources that fish are adapted to consume. Previous studies have proven that the inclusion of small amounts (<10% of the diet) of algae in fish feed (aquafeed) resulted in positive effects in growth performance and feed utilisation efficiency. Marine algae have also been shown to possess functional activities, helping in the mediation of lipid metabolism, and therefore are increasingly studied in human and animal nutrition. The aim of this study was to assess the potentials of two commercially available algae derived products (dry algae meal), Verdemin (derived from *Ulva ohnoi*) and Rosamin (derived from diatom *Entomoneis* spp.) for their possible inclusion into diet of Atlantic Salmon (*Salmo salar*). Fish performances, feed efficiency, lipid metabolism and final product quality were assessed to investigated the potential of the two algae products (in isolation at two inclusion levels, 2.5% and 5%, or in combination), in experimental diets specifically formulated with low fish meal and fish oil content. The results indicate that inclusion of algae product Verdemin and Rosamin at level of 2.5 and 5.0% did not cause any major positive, nor negative, effect in Atlantic Salmon growth and feed efficiency. An increase in the omega-3 long-chain polyunsaturated fatty acid (n-3 LC-PUFA) content in whole body of fish fed 5% Rosamin was observed.

## Introduction

Fish meal and fish oil are abundantly used in fish feed (aquafeed) largely due to their respective content of high-quality proteins and beneficial omega-3 long chain polyunsaturated fatty acid (n-3 LC-PUFA). A global survey estimated aquaculture consumption of fish meal and fish oil at above 4,000 and 800 thousand tonnes/year, equating to 68.2% and 88.5% of the yearly global supply, respectively [1]. However, because of their limited supply and raising prices, alternative raw materials are increasingly being used in aquafeed formulation. A critical shortcoming of the crop-plant derived protein sources commonly used in aquafeed is that they have low digestibility and are deficient in certain essential amino acids such as lysine, methionine, threonine and tryptophan [2]; whereas for terrestrial oils the major limit is their lack of omega-3 long chain polyunsaturated fatty acid (n-3 LC-PUFA) [3]. Therefore it is important to find economical and sustainable alternative sources of proteins and lipids [4], and algae-derived products may be one possible alternative feedstuff, because of their nutritional quality and potential availability.

Analysis of amino acid content of numerous algae have found that although there is significant variation in total protein content (8–50% dry weight) [5], they generally contain all the essential amino acids [5–8]; and studied algae have also been found to be rich in n-3 LC-PUFA; essentially algae derived products seem to be a very suitable raw material for use in aquafeed [9]. Algae are at the base of the aquatic food chain that produce the food resources that fish are adapted to consume. Accordingly, since the ‘70s, the first pioneering studies focusing on the potential use of algae in aquafeed were published [10]. However, more recently, increasingly more studies have been carried out on the use of algae as a possible ingredient for aquafeed for different fish species [11–20].

Several studies have proven that small amounts (2.5–10% of the diet) of algae in fish diets resulted in positive effects, including: increase in growth performance, feed utilisation efficiency, carcass quality, physiological activity, intestinal micro biota (Valente et al 2006, Mustafa and Nakagawa, 1995), disease resistance [21], improved stress response [22], modulation of the lipid metabolism [12,23], and improved protein retention during the winter period of reduced feed intake [24], and increase palatability in sea urchin formulated feed [25]. Algae meal has been also recommended as a good feed complement for counteracting intestinal inflammation produced by soybean meal [20], and as a useful binding agent for aquafeed pelletisation [19].

Nonetheless, it has also been noted that the use of algae in aquafeed at a high inclusion level might have a negative effect on fish growth and feed efficiency. Previous studies in rainbow trout (*Oncorhynchus mykiss*) showed a reduction in growth and feed utilisation in fish feed with 10% of *Ulva* spp. and similar results were found in black sea bream (*Acanthopagrus schlegeli*) [12] and gilthead sea bream (*Sparus aurata*) [22] fed diets containing 15% ulva meal, and in common carp (*Cyprinus carpio*) [15] and Nile tilapia (*Oreochromis niloticus*) fed with diets containing 20% algae meal (*Ulva rigida*) [26]. These results could be due to certain substances with anti-nutritional activity which may be present in algae, like lectins, tannins, phytic acid, and protease and amylase inhibitors [27].

From the literature available, it can be deduced that the response to algae inclusion into feed is dose-dependent and species-specific [16]. In mammals, it has been shown that marine algae, and particularly *Ulva* spp. [28], have potential beneficial effects as dietary antioxidants [29,30], and because of their bioactive properties. Specifically, functional properties of algae polysaccharides [5] and fucoxanthin (a marine carotenoid present in brown algae and diatoms) [31,32] have been shown to be important mediators in lipid metabolism [32–34], and are increasingly studied in human and animal nutrition. Thus, because of this series of potential highly bioactive compounds, algae derived products could be considered as a very valuable/useful “micro-ingredient” for aquafeed fortification, when used at a low inclusion level.

The aim of this study was to assess the potentials of two commercially available algae derived products, and specifically a green algae (Verdemin, derived from macro algae *Ulva ohnoi*) and a diatom, (Rosamin, derived from diatom *Entomoneis* spp.) for their possible inclusion into diet of Atlantic Salmon (*Salmo salar*). Fish performances, feed efficiency, lipid metabolism and final product quality were assessed to investigate the potential of the two algae products, which were tested in solely at two inclusion levels (2.5% and 5%), or in combination, in experimental diets specifically formulated (with low fish meal content) for juvenile Salmon.

## Materials and Methods

### Fish husbandry and experimental diets

All procedures implemented during this experiment were approved by the Deakin University Animal Ethics Committee (AEC ref B16-2013). Atlantic Salmon (*Salmo salar*) were sourced from a private aquaculture farm (Mountain Fresh Trout and Salmon Farm, Harrietville, VIC, Australia). After transport, fish were acclimatised to experimental conditions at Deakin University (Warrnambool, VIC, Australia) and fed on a commercial diet (Ridley Aquafeed, Australia) for two weeks. At the start of the experiment, 10 fish were euthanized with an overdose of anaesthetic (AQUI-S, New Zealand), weighed and stored at -20°C, until analysis.

Five hundred and forty fish (body weight ~33.7 g) were anaesthetised, weighed and then randomly distributed into 18 tanks (30 fish per tank) of 1000 L fresh water capacity, within a fully controlled multi-tank recirculation system (RAS), and three tanks were randomly assigned to each of the six dietary treatments. Fish were held at 12°C, under 12:12 light: dark cycle, and total ammonia and nitrite levels were regularly monitored using Aquamerck test kits (Merck, Germany) and were maintained within optimal levels. Fish were fed the respective experimental diet twice daily to apparent satiation for 84 days. Then, after a 24 h gut evacuation period, all fish were anaesthetised and weighed. A final sample of 6 fish per tank (18 per treatment) was randomly collected and euthanised, and samples of fillet and liver (3 fish per tank) or the whole body (3 fish per tank) were collected and stored at -20°C, until proximate and fatty acid analysis.

Six iso-proteic, iso-lipidic and iso-energetic experimental diets were formulated to contain 180 mg/g of lipid and 490 mg/g of protein, varying only in in the inclusion of two algal products Verdemin (derived from *Ulva ohnoi*) and Rosamin (derived from diatom *Entomoneis* spp.) (Table 1). All diets contained the same blend of fish oil (25%) and poultry oil (75%) as the added lipid source, and the same blend of protein sources, including fish meal (15%), soy protein concentrate, gluten, poultry meal, whey and blood meal were used. The diet without any algal inclusion, and with a formulation similar to commercially available Salmon diets, was considered the control diet (CD). In the 5 remaining experimental diets Verdemin and Rosamin were included solely at two different levels (2.5% or 5%) or in combination, both at 2.5%. Accordingly, the experimental diets were named: LV (Low Verdemin; at 2.5%); HV (High Verdemin; at 5%); LR (Low Rosamin; at 2.5%); HR (High Rosamin; at 5%); and VR (Verdemein at 2.5% and Rosamin at 2.5%). To compensate for the resulting modification of the overall nutritional value of the experimental diets due to algal addition, small and balanced amounts of poultry meal, poultry oil, blood meal, pregelatinised starch and wheat flour were used (Table 1), so that final diets would have been iso-proteic, iso-lipidic and iso-energetic; but the total inclusion of fish meal and fish oil was not modified.

**Table 1:** Formulation and proximate composition of the diets.

	*Experimental treatment* ^*1*^					
* *	CD	LV	HV	LR	HR	VR
*Diet formulation (mg/g)*						
Fish meal^2^	150	150	150	150	150	150
Soy protein concentrate^2^	102	102	102	102	102	102
Soybean meal^2^	67	67	67	67	67	67
Wheat gluten^3^	53	53	53	53	53	53
Whey^4^	45	45	45	45	45	45
Fish oil^2^	37.5	37.5	37.5	37.5	37.5	37.5
Vitamin & mineral mix^5^	7.0	7.0	7.0	7.0	7.0	7.0
Celite^6^	5.0	5.0	5.0	5.0	5.0	5.0
Meat and bones meal^7^	5.0	5.0	5.0	5.0	5.0	5.0
Choline^2^	4.9	4.9	4.9	4.9	4.9	4.9
Monosodium phosphate^2^	4.0	4.0	4.0	4.0	4.0	4.0
Lysine^2^	1.5	1.5	1.5	1.5	1.5	1.5
Methionine^2^	1.0	1.0	1.0	1.0	1.0	1.0
Taurine^2^	1.0	1.0	1.0	1.0	1.0	1.0
Vitamin C^2^	0.5	0.5	0.5	0.5	0.5	0.5
Pigment^2^ (carophil pink)	0.06	0.06	0.06	0.06	0.06	0.06
*(Raw materials – adjusted)*						
Poultry meal^2^	195	195	195	191	188	191
Poultry oil^2^	116	116	116	114	113	114
Blood meal^2^	92	88	83	92	91	87
Pregel Starch^2^	76	59	43	67	59	51
Wheat flour^2^	38	34	30	27	16	23
*(Algal products)*						
Rosamin^8^	0	0	0	25	50	25
Verdemin^9^	0	25	50	0	0	25
*Proximate composition*						
Moisture (mg/g)	67	78	44	71	99	99
Protein (mg/g)	492	483	499	487	475	472
Lipid (mg/g)	186	184	186	184	185	183
Ash (mg/g)	79	83	90	88	97	91
N.F.E.^10^ (mg/g)	176	177	181	169	143	154
Energy^11^ (KJ/g)	22	21	22	21	21	21

^1^ Experimental diet abbreviations: CD (Control Diet), LV (2.5% Verdemin), HV (5.0% Verdemin), LR (2.5% Rosamin), HR (5.0% Rosamin) and VR (2.5% Verdemin and 2.5% Rosamin).

^2^ Ridley Agriproducts, Narangba, Queensland, Australia.

^3^ Agrifood Ingredients, Kew East, Victoria Australia.

^4^Warrnambool Cheese and Butter, Victoria, Australia.

^5^ DSM Nutritional Products,WaggaWagga, New SouthWales, Australia.

^6^ Merck KGaA, Darmstadt, Germany.

^7^ The Midfield Group,Warrnambool, Victoria, Australia.

^8^ Rosamin: *Entomoneis* spp. dry meal (MBD Energy ltd. Melbroune, Victoria, Australia): Moisture 88.1 mg/g; Protein 190.1 mg/g; Lipid 4.4 mg/g; Ash 221.6 mg/g.

^9^ Verdemin: *Ulva ohnoi* dry meal (MBD Energy ltd. Melbroune, Victoria, Australia): Moisture 22.8 mg/g; Protein 192.3 mg/g; Lipid 93.3 mg/g; Ash 483.0 mg/g.

^10^ N.F.E. = Nitrogen free extract, calculated by difference.

^11^ Calculated on the basis of 23.6, 39.5 and 17.2 kJ/g of protein, fat and carbohydrate, respectively.

The experimental diets were specifically formulated with the inclusion of a source of acid insoluble ash (Celite) as internal marker for digestibility evaluation, and were then manufactured and pelletised using a small scale extruder (DGP-50, Zhengzhou Amisy Machinery Co.ltd, Zhengzhou, Henan, China). Briefly, all ingredients were combined in a commercial baker's mixer (MEC, Victoria, Australia) and mixed thoroughly, before addition of 2–3 l of water (at 80°C) per 20 kg of diet and further mixed. The mash was then loaded on the extruder preconditioning mixer hopper that automatically fed the single-screw extruder, fitted with a variable speed cutter, obtaining pellet size of 5 mm diameter m and 5 mm length. Finished pellets were then dried in a temperature controlled fan-forced room at 40°C over a 12-h period, and stored at -20° C in airtight bags until needed.

### Performance parameters and chemical analyses

Standard formulae were used to assess growth and feed utilisation parameters over the experimental period, and were computed as previously described [35]; these included initial and final average weight (g), average feed consumption (g fish^-1^), gain in weight (g and %), food conversion ratio (FCR), specific growth rate (SGR, % day^-1^), feed ratio (% of body weight), dress-out percentage (DP%), fillet yield percentage (FY%), hepatosomatic index (HSI%) and condition factor (K).

The chemical composition of the experimental diets, faeces and fish samples was determined via proximate composition analysis according to standard methods [36]. Lipid was determined by dichloromethane:methanol extraction (2:1) technique [37], with the substitution of chloroform with dichloromethane for safety reasons and the addition of butylated hydroxytoluene (BHT) (50 mg L^-1^) to reduce lipid oxidation during processing. After lipid extraction, an aliquot was used for fatty acid analysis, which was implemented via trans-methylation and gas chromatography, following the procedures previously described in detail [38].

### Nutrient Digestibility and fatty acid metabolism evaluation

During days 53 to 84, faeces were collected from each individual tank using a previously described method [39]. Nutrients apparent digestibility coefficients were determined by assessing acid insoluble ash (AIA), as specifically adapted to rainbow trout [40], and following standard formulae for digestibility evaluation.

The evaluation of the *in vivo* fatty acid metabolism (fatty acid apparent in vivo β-oxidation, bioconversion and deposition) was performed using the whole-body fatty acid balance method, as initially proposed and described [41], with further development [42].

### Statistical analysis

All data were reported as mean ± standard error (*n* = 3, *N* = 18). After confirmation of normality and homogeneity of variance, data was first subjected to a one-way ANOVA, with significance accepted at *P* < 0.05. Where significant differences were detected by ANOVA, data was subjected to a Student–Newman–Keuls Post-hoc test for homogenous subsets. All statistical analyses were performed using IBM SPSS Statistics v21.0 (SPSS Inc.,Chicago, IL, USA).

## Results

### Diets and fish performances

Experimental diets were iso-proteic, iso-lipidic and iso-energetic (Table 1), and their fatty acid composition was almost identical (Table 2). All experimental diets were readily accepted by fish, and overall fish showed optimal growth performances, minimal mortality and good feed conversion (low FCR; varying from 0.77 to 0.86) (Table 3). HR fed fish recorded a significantly higher feed intake compared to HV, but fish feed with HV showed a significantly lower (better) FCR compared to HR. The dietary treatment did not affect any biometrical parameter recorded on fish, including DP, FY, HSI and K (Table 3). With the exception of feed intake and FCR for HR and VR, none of the other performances, nutrient efficiency and biometrical parameters showed any statistically significant difference among dietary treatments.

**Table 2:** The fatty acid composition (% of total fatty acids) and total fatty acid content (mg/g of lipid) of the six experimental diets and the two tested algal products.

	*Experimental treatments* ^*1*^						*Algal products*	
*% of total fatty acids*	CD	LV	HV	LR	HR	VR	Verdemin	Rosamin
12:00	0.14	0.13	0.07	0.13	0.08	0.08	-	0.33
14:00	2.91	2.9	2.9	3.01	3.18	3.01	-	22.7
14:1n-5	0.26	0.26	0.26	0.26	0.3	0.3	-	-
16:00	23.74	23.72	23.76	23.66	25.03	25.15	49.94	17.51
16:1n-7	6.35	6.36	6.37	6.46	7.27	7.17	-	27.99
17:00	0.32	0.32	0.32	0.31	0.32	0.3	-	-
17:1n-7	0.18	0.18	0.19	0.18	0.19	0.19	-	-
18:00	6.61	6.42	6.4	6.43	6.56	6.56	-	0.47
18:1n-9	36.12	36.19	36.18	36.02	34.67	34.91	9.03	0.91
18:1n-7	2.7	2.69	2.71	2.71	2.81	2.81	10.9	1.25
18:2n-6	10.41	10.51	10.52	10.37	9.41	9.51	7.08	0.95
18:3n-6	0.18	0.18	0.18	0.18	0.18	0.18	-	0.77
18:3n-3	1.46	1.47	1.47	1.45	1.08	1.11	9.77	0.31
20:00	0.22	0.21	0.22	0.23	0.23	0.24	-	0.42
18:4n-3	0.6	0.6	0.61	0.62	0.6	0.59	11.13	0.42
20:1n-13	0.09	0.09	0.09	0.07	0.09	0.09	-	-
20:1n-11	0.09	0.09	0.09	0.08	0.11	0.1	-	-
20:1n-9	0.54	0.55	0.54	0.53	0.57	0.54	-	-
20:2n-6	0.13	0.13	0.13	0.13	0.14	0.13	-	-
20:3n-6	0.11	0.12	0.11	0.12	0.07	0.13	-	0.18
20:4n-6	0.38	0.38	0.38	0.4	0.42	0.41	0.43	6.49
20:3n-3	0.03	0.03	0.03	0.03	0.03	0.03	-	0.19
22:00	0.07	0.08	0.08	0.08	0.08	0.08	-	-
20:4n-3	0.21	0.21	0.21	0.25	0.23	0.27	-	-
22:1n-11	0.12	0.13	0.13	0.13	0.14	0.14	-	-
22:1n-9	0.06	0.06	0.06	0.07	0.07	0.07	-	-
20:5n-3	3.56	3.56	3.54	3.65	3.69	3.48	1.64	16.74
22:2n-6	-1	-	-	-	-	-	-	-
22:4n-6	0.05	0.05	0.05	0.05	0.06	0.07	-	1.3
22:5n-6	0.06	0.06	0.06	0.06	0.06	0.06	-	-
24:1n-9	0.12	0.12	0.12	0.13	0.13	0.12	-	-
22:5n-3	0.43	0.44	0.45	0.45	0.45	0.45	-	-
22:6n-3	1.73	1.74	1.74	1.75	1.76	1.71	0.07	1.06
24:5n-3	-	-	-	-	-	-	-	-
24:6n-3	-	-	-	-	-	-	-	-
SFA^2^	34.01	33.78	33.76	33.85	35.47	35.42	49.94	41.43
MUFA^3^	46.64	46.74	46.74	46.64	46.35	46.44	19.93	30.14
PUFA^4^	19.35	19.49	19.5	19.51	18.17	18.14	30.13	28.4
n-6 PUFA^5^	11.33	11.43	11.44	11.31	10.34	10.5	7.51	9.69
n-6 LC-PUFA^6^	0.73	0.74	0.74	0.77	0.75	0.8	0.43	7.97
n-3 PUFA^7^	8.03	8.06	8.06	8.19	7.84	7.65	22.62	18.7
n-3 LC-PUFA^8^	5.97	5.98	5.98	6.13	6.15	5.95	1.71	17.98
n-3/n-6 ratio^9^	0.094	0.094	0.097	0.10	0.10	0.73	3.01	1.93
Total Fatty acids^10^ (mg/g)	755.8	746.2	728.8	727.6	740.6	721.6	801	817.5

^1^ abbreviation as in Table 1.

^2^ - = not detected.

^3^ SFA = Saturated fatty acids.

^4^ MUFA = monounsaturated fatty acids.

^5^ PUFA = polyunsaturated fatty acids.

^6^ n-6 PUFA = omega-6 polyunsaturated fatty acids.

^7^ n-6 LC-PUFA = long chain omega-6 polyunsaturated fatty acids.

^8^ n-3 PUFA = omega-3 polyunsaturated fatty acids.

^9^ n-3 LC-PUFA = long chain omega-3 polyunsaturated fatty acids.

^10^ n-3/n-6 ratio = ratio of n-3 PUFA/n-6 PUFA.

**Table 3:** Fish performances and feed efficiency in Atlantic Salmon fed the six experimental diets during the in vivo feeding trial.

	Experimental treatments ^1^					
	CD	LV	HV	LR	HR	VR
Init. Wt (g/fish)	33.7±0.3	34.3±1.2	33.6±0.3	33.6±0.7	33.7±0.3	33.6±0.1
Final Wt (g/fish)	137.0±2.2	131.7±2.0	132.2±0.7	134.7±0.8	132.7±1.1	134.0±1.8
Survival (%)	97.8±2.2	100	100	97.8±2.2	95.6±4.4	97.8±2.2
Feed intake (g/fish)	81.9±1.5	79.3±1.5	76.3±1.1	80.4±1.1	85.2±2.8	82.6±2.8
Feed intake (% bw/day)	1.17±0.02^ab^	1.17±0.00^ab^	1.12±0.01^a^	1.16±0.02^ab^	1.25±0.05^b^	1.20±0.03^ab^
Gain (g/fish)	103.3±2.3	97.4±1.0	98.6±0.6	101.1±1.4	99.0±1.1	100.5±2.0
Gain (% init. Wt)	306.5±8.7	285.0±8.3	293.6±2.9	301.0±10.3	294.1±4.4	299.4±6.9
FCR^2^	0.79±0.01^ab^	0.81±0.01^ab^	0.77±0.01^a^	0.79±0.00^ab^	0.86±0.04^b^	0.82±0.01^ab^
SGR^3^ (%/day)	1.71±0.03	1.64±0.03	1.67±0.01	1.69±0.03	1.67±0.01	1.69±0.02
PER^4^	2.56±0.03	2.54±0.03	2.59±0.02	2.58±0.00	2.45±0.10	2.58±0.04
PGR^5^ (%/day)	1.87±0.03	1.78±0.02	1.85±0.01	1.86±0.02	1.83±0.02	1.83±0.02
%NPU^6^	51.1±0.7	49.4±0.6	52.9±0.3	51.9±1.4	48.9±2.8	50.3±1.3
FDR^7^ (%/day)	1.68±0.01	1.59±0.06	1.56±0.01	1.59±0.08	1.52±0.02	1.58±0.08
DP%^8^	90.6±0.1	91.1±0.1	90.8±0.4	91.0±0.2	91.2±0.2	91.0±0.1
FY%^9^	55.5±1.2	54.9±0.7	55.0±1.4	54.0±1.1	52.0±0.1	52.9±2.1
HSI%^10^	1.0±0.1	1.0±0.0	1.0±0.0	1.0±0.0	1.1±0.0	1.0±0.0
K^11^	1.0±0.0	1.0±0.0	1.0±0.0	1.0±0.0	1.0±0.0	1.0±0.0

Data are expressed as mean ± S.E.M., n = 3; N = 18. Values in the same row with different letters (a, b, c) are significantly different (P < 0.05; ANOVA and Student–Newman–Keuls post hoc test).

^1^ abbreviation as in Table 1.

^2^ FCR = food conversion ratio.

^3^ SGR = specific growth rate.

^4^ PER = protein efficiency ratio.

^5^ PGR = protein growth rate.

^6^%NPU = net protein utilisation.

^7^ FRD = fat deposition rate.

^8^ DP% = dress-out percentage.

^9^ FY% = Fillet yield percentage.

^10^ HSI% = Hepato-Somatic index.

^11^ K = Fulton’s condition factor.

No differences in fillet and whole body nutritional value and body composition were observed among dietary treatments. However, diets with highest Rosamin inclusion showed a partial (not statistically significant) reduction in total lipid content in both fillet and whole body analysis (Table 4).

**Table 4:** The proximate composition (mg/g) of fillets and whole bodies of Atlantic Salmon at commencement and the completion of the in vivo feeding trial.

		Experimental treatments ^1^					
	*initial*	CD	LV	HV	LR	HR	VR
*Fillet*							
Moisture	750.7±6.3	729.4±2.5	726.6±5.0	729.4±1.5	723.6±4.2	729.3±1.6	730.7±4.4
Protein	201.8±5.2	216.5±2.6	214.1±3.4	216.4±0.9	220.5±2.8	217.6±1.0	212.3±3.2
Lipid	34.1±2.2	40.5±3.6	46.1±1.3	41.4±2.6	42.3±2.3	39.4±0.7	43.6±3.1
Ash	13.4±0.4	13.6±0.3	13.2±0.7	12.7±0.4	13.6±0.7	13.7±0.2	13.5±0.3
*Whole body*							
Moisture	715.3±1.8	700.0±0.9	705.4±1.9	702.5±1.7	703.8±1.2	709.0±4.3	708.6±3.9
Protein	168.1±3.9	191.6±1.1	187.6±1.8	194.9±0.6	192.7±3.8	191.1±2.9	188.4±2.0
Lipid	93.1±2.1	90.9±1.8	89.6±3.2	85.3±1.4	86.0±3.9	82.4±1.2	85.4±5.2
Ash	23.4±0.5	17.4±0.5	17.5±0.6	17.3±0.3	17.5±0.7	17.6±0.2	17.6±0.6

Initial fish not included in statistical analysis. Data are expressed as mean ± S.E.M., n = 3; N = 18, no statistically significant differences between treatments for any parameter were observed by ANOVA.

^1^ abbreviation as in Table 1.

### Nutrients digestibility

Nutrients apparent digestibility was significantly affected by dietary algal inclusion (Table 5). Specifically, the protein apparent digestibility coefficient (ADC%) was lower in high Verdemin inclusion (HV) compared to all other treatments. A similar trend was observed in dry matter digestibility with the lowest value recorded in HV, however this was not significant.

**Table 5:** The nutrients apparent digestibility coefficients (ADC%) in fish fed the six experimental diets.

	Experimental treatments ^1^					
	CD	LV	HV	LR	HR	VR
ADC% Dry matter	71.8±0.9	68.5±0.7	64.1±3.0	70.5±0.9	69.2±1.1	68.2±1.1
ADC% Protein	86.1±0.4^c^	83.1±0.7^b^	80.6±0.8^a^	84.6±0.6^bc^	82.8±0.5^b^	83.3±0.5^b^
ADC% Lipid	87.8±1.0	87.4±0.8	86.0±0.8	87.8±0.5	87.2±0.7	85.7±0.8
ADC% NFE	32.1±2.7	26.6±1.5	16.5±9.8	30.7±2.1	24.0±4.8	21.8±2.4

Data are expressed as mean ± S.E.M., n = 3; N = 18. Values in the same row with different letters (a, b, c) are significantly different (P < 0.05; ANOVA and Student–Newman–Keuls post hoc test).

^1^ abbreviation as in Table 1.

Several statistically significant differences were observed for digestibility of individual fatty acid (Table 6). Summarising the majority of the observed differences, it can be noted that the three treatments containing up to 5% of algal product (HV, HR and VR) resulted in lower fatty acid digestibility, compared to the other three treatments. CD, generally was responsible for the highest individual fatty acid digestibility, with the exception of n-6 LC-PUFA which were more efficiently digested in fish fed LR. The inclusion of 5% Verdemin (HV) resulted in reduced digestibility of 20:5n-3 (EPA) and 22:6n-3 (DHA).

**Table 6:** The individual fatty acid and fatty acid classes apparent digestibility coefficients (ADC%) in fish fed the six experimental diets.

* *	*Experimental treatments* ^*1*^					
*ADC (%)*	CD	LV	HV	LR	HR	VR
12:0	90.2±2.9^ab^	91.1±3.6^ab^	74.5±7.6^a^	93.8±1.3^b^	90.2±0.8^ab^	90.0±0.3^ab^
14:0	86.7±1.4	84.4±0.9	85.3±2.2	84.3±0.8	85.9±0.7	83.2±1.1
14:1n-5	95.1±0.5	95.1±0.3	93.7±0.7	94.3±0.5	94.9±0.1	94.3±0.4
16:0	78.5±2.3	76.8±1.5	74.7±1.5	77.0±1.3	78.1±1.3	74.6±1.8
16:1n-7	96.6±0.2	96.8±0.1	95.9±0.3	96.8±0.1	96.3±0.1	96.0±0.1
17:0	77.3±2.1	74.4±1.1	71.1±1.8	72.3±2.0	77.1±2.0	70.5±2.3
17:1n-7	95.1±0.1	94.7±0.6	93.9±0.3	94.3±0.3	94.1±0.3	94.6±0.6
18:0	71.1±3.4	70.2±2.1	67.5±1.6	71.3±1.9	73.6±2.1	68.0±2.4
18:1n-9	94.8±0.3	95.1±0.2	94.3±0.5	95.1±0.2	94.1±0.3	93.8±0.1
18:1n-7	93.4±0.4	93.2±0.2	91.9±0.7	93.3±0.2	92.2±0.3	91.8±0.2
18:2n-6	96.6±0.2^ab^	96.8±0.1^b^	95.9±0.3^a^	96.8±0.1^b^	96.1±0.2^ab^	95.9±0.0^a^
18:3n-6	95.2±0.2	95.1±0.3	93.5±1.2	94.9±0.3	94.2±0.1	94.1±0.1
18:3n-3	97.2±0.2^b^	97.3±0.1^b^	96.4±0.2^a^	97.2±0.2^b^	96.4±0.2^a^	96.1±0.2^a^
20:0	78.3±2.8	75.1±1.7	72.5±1.3	78.4±1.7	77.3±1.2	74.7±1.9
18:4n-3	98.9±0.0^bc^	98.9±0.1^bc^	98.1±0.1^a^	99.1±0.1^c^	99.0±0.1^c^	98.6±0.0^b^
20:1n-13	99.1±0.9^b^	99.0±1.0^b^	98.1±1.9^b^	100	91.9±0.9^a^	89.8±1.7^a^
20:1n-11	93.6±0.7	87.9±6.4	96.8±1.7	93.5±0.1	95.6±2.2	93.8±0.5
20:1n-9	91.7±0.6	90.9±1.2	92.1±0.9	91.9±0.2	93.6±2.3	90.0±0.5
20:2n-6	82.3±1.3	81.7±1.4	77.9±1.1	83.0±1.1	83.1±0.7	77.6±2.0
20:3n-6	83.6±1.6	81.4±3.2	80.1±1.0	88.6±3.7	79.3±2.9	83.7±3.9
20:4n-6	96.3±0.2^bc^	96.2±0.1^bc^	94.8±0.4^a^	96.5±0.1^c^	96.1±0.2^bc^	95.5±0.1^b^
20:3n-3	100^2^	100	100	100	100	100
22:0	60.6±4.4	54.4±3.7	47.2±2.3	59.1±3.8	60.7±1.4	51.2±3.6
20:4n-3	95.7±0.6	94.3±1.4	95.3±0.7	96.9±0.3	95.6±0.5	94.7±0.3
22:1n-11	89.1±0.8^ab^	88.8±0.8^ab^	87.1±0.9^a^	90.4±0.2^b^	89.1±0.5^ab^	87.8±0.2^ab^
22:1n-9	81.0±1.2^ab^	78.9±1.6^ab^	76.5±1.0^a^	83.4±0.8^b^	81.8±0.6^b^	79.3±1.4^ab^
20:5n-3	98.4±0.1^c^	98.5±0.0^c^	97.7±0.2^a^	98.5±0.0^c^	98.2±0.1^bc^	98.0±0.0^ab^
22:2n-6	n.d.^3^	n.d.	n.d.	n.d.	n.d.	n.d.
22:4n-6	96.3±3.7	96.3±1.9	89.0±2.6	97.5±2.5	90.4±0.4	90.5±0.4
22:5n-6	98.9±1.1	100	100	100	98.1±1.9	100
24:1n-9	76.7±2.0^b^	72.6±1.8^ab^	67.4±1.3^a^	72.1±2.1^ab^	74.1±0.6^ab^	67.0±2.1^a^
22:5n-3	97.6±0.1^b^	97.7±0.0^b^	96.7±0.3^a^	97.6±0.2^b^	97.3±0.2^b^	97.0±0.1^ab^
22:6n-3	96.8±0.2^b^	96.8±0.1^b^	95.6±0.4^a^	97.0±0.0^b^	96.8±0.1^b^	96.3±0.1^b^
24:5n-3	n.d.	n.d.	n.d.	n.d.	n.d.	n.d.
24:6n-3	n.d.	n.d.	n.d.	n.d.	n.d.	n.d.
SFA^4^	77.8±2.4	76.2±1.6	74.2±1.4	76.5±1.4	77.9±1.4	74.0±1.8
MUFA^4^	94.9±0.3	95.1±0.2	94.2±0.4	95.1±0.2	94.2±0.3	93.8±0.1
PUFA^4^	96.9±0.2^b^	97.0±0.1^b^	96.0±0.3^a^	97.1±0.1^b^	96.5±0.2^a^b	96.2±0.0^a^
n-6 PUFA^4^	96.3±0.2^b^	96.5±0.1^b^	95.4±0.3^a^	96.5±0.1^b^	95.8±0.2^ab^	95.4±0.1^a^
n-6 LC-PUFA^4^	92.0±0.9^ab^	91.5±0.6^ab^	89.5±0.5^a^	93.3±0.6^b^	92.0±0.4^ab^	90.6±1.0^ab^
n-3 PUFA^4^	97.7±0.1^c^	97.8±0.1^c^	96.9±0.2^a^	97.9±0.1^c^	97.5±0.1^bc^	97.2±0.0^ab^
n-3 LC-PUFA^4^	97.8±0.1^c^	97.8±0.1^c^	96.9±0.2^a^	97.9±0.0^c^	97.6±0.1^bc^	97.2±0.0^ab^
Total Fatty Acids	89.5±1.0	89.1±0.6	87.8±0.7	89.2±0.4	88.9±0.5	87.3±0.7

Data are expressed as mean ± S.E.M., n = 3; N = 18. Values in the same row with different letters (a, b, c) are significantly different (P < 0.05; ANOVA and Student–Newman–Keuls post hoc test).

^1^ abbreviation as in Table 1.

^2^ADC% = 100%, it means that the fatty acid was not detected in the faeces.

^3^ADC% = n.d., it means that the fatty acid was not detected in the feed.

^4^ abbreviation as in Table 2.

### Fatty acid composition

A series of statistically significant differences were recorded for fatty acid composition of fish tissues (whole body and fillet; Table 7 and Table 8, respectively), and overall similar trends and/or differences relative to dietary treatments were observed in both tissues. Fish fed diets containing Rosamin (LR and HR) recorded a significantly lower level of 16:0, in respect to fish on CD. This also resulted in significantly lower total SFA content in the whole body. Some significant differences were also recorded for a few MUFA, such as 14:1n-5, 16:1n-7 and 18:1n-7, with fish receiving diets containing Rosamin, in isolation at both inclusion levels (LR and HR), and also in combination with Verdemin (VR), generally showing higher levels of these fatty acids, compared to other treatments. Linoleic acid (18:2n-6) and alpha-linolenic acid (18:3n-3) were also affected by dietary treatments, with lower levels of both being recorded in fish fed HR and VR, compared to other treatments. This resulted in lower n-6 PUFA content in the fillets of fish fed HR and VR.

**Table 7:** The fatty acid composition (% of total fatty acids) and total fatty acid content (mg/g of lipid) of the whole body of Atlantic Salmon at commencement and the completion of the in vivo feeding trial (initial fish not included in statistical analysis; see Table 3 for fatty acid classes’ nomenclature).

		*Experimental treatments* ^*1*^					
*% of total fatty acids*	*Initial*	CD	LV	HV	LR	HR	VR
12:0	*0*.*08*	0.08±0.00	0.09±0.00	0.09±0.00	0.09±0.00	0.08±0.00	0.08±0.00
14:0	*4*.*33*	2.68±0.07	2.76±0.01	2.74±0.02	2.82±0.01	2.85±0.04	2.77±0.03
14:1n-5	*0*.*2*	0.15±0.02^a^	0.19±0.00^b^	0.19±0.00^b^	0.19±0.00^b^	0.21±0.00^b^	0.20±0.01^b^
16:0	*17*.*67*	18.05±0.53	18.48±0.06	18.58±0.11	18.16±0.11	18.32±0.17	18.67±0.11
16:1n-7	*7*.*68*	6.04±0.16^a^	6.32±0.03^a^	6.26±0.02^a^	6.45±0.02^ab^	7.08±0.04^c^	6.79±0.22^bc^
17:0	*0*.*36*	0.27±0.01	0.28±0.00	0.28±0.00	0.27±0.01	0.27±0.01	0.28±0.00
17:1n-7	*0*.*24*	0.20±0.00	0.21±0.00	0.19±0.01	0.21±0.00	0.21±0.00	0.21±0.00
18:0	*4*.*75*	5.30±0.13	5.42±0.06	5.52±0.04	5.25±0.04	5.25±0.05	5.36±0.06
18:1n-9	*27*.*94*	37.81±1.46	35.63±0.09	35.50±0.03	35.77±0.21	35.06±0.21	35.37±0.06
18:1n-7	*3*.*7*	3.37±0.07^a^	3.48±0.03^ab^	3.52±0.04^ab^	3.57±0.02^bc^	3.72±0.03^c^	3.60±0.06^bc^
18:2n-6	*7*.*88*	9.67±0.00^ab^	9.99±0.04^c^	9.88±0.04^bc^	10.06±0.06^c^	9.43±0.07^a^	9.55±0.16^a^
18:3n-6	*0*.*28*	0.31±0.01	0.33±0.00	0.34±0.01	0.33±0.01	0.34±0.02	0.34±0.00
18:3n-3	*1*.*21*	1.09±0.02^bc^	1.17±0.01^c^	1.15±0.01^c^	1.15±0.01^c^	0.98±0.00^a^	1.02±0.06^ab^
20:0	*0*.*18*	0.18±0.00	0.17±0.00	0.18±0.01	0.17±0.00	0.18±0.01	0.18±0.01
18:4n-3	*1*.*2*	0.62±0.02	0.66±0.01	0.67±0.01	0.65±0.01	0.65±0.01	0.66±0.01
20:1n-13	*0*.*36*	0.24±0.01^a^	0.26±0.01^a^	0.25±0.00^a^	0.25±0.00^a^	0.29±0.01^b^	0.26±0.01^a^
20:1n-11	*0*.*11*	0.09±0.00	0.10±0.00	0.09±0.00	0.09±0.00	0.11±0.01	0.10±0.00
20:1n-9	*1*.*21*	1.45±0.04	1.48±0.02	1.46±0.01	1.47±0.04	1.43±0.01	1.45±0.01
20:2n-6	*0*.*46*	0.74±0.02	0.77±0.01	0.77±0.01	0.77±0.01	0.72±0.01	0.74±0.00
20:3n-6	*0*.*3*	0.48±0.02	0.51±0.01	0.51±0.01	0.51±0.01	0.49±0.01	0.50±0.01
20:4n-6	*0*.*83*	0.63±0.02^a^	0.66±0.01^ab^	0.68±0.00^b^	0.69±0.01^b^	0.71±0.00^b^	0.69±0.01^b^
20:3n-3	*0*.*09*	0.10±0.00^a^	0.11±0.00^ab^	0.11±0.00^ab^	0.11±0.00^b^	0.10±0.00^a^	0.10±0.00^ab^
22:0	*0*.*1*	0.11±0.02	0.07±0.00	0.07±0.00	0.07±0.00	0.07±0.01	0.07±0.00
20:4n-3	*0*.*6*	0.37±0.01	0.40±0.01	0.40±0.01	0.39±0.01	0.40±0.00	0.39±0.00
22:1n-11	*0*.*36*	0.17±0.01	0.17±0.00	0.17±0.00	0.17±0.00	0.18±0.01	0.18±0.00
22:1n-9	*0*.*16*	0.15±0.00	0.15±0.00	0.15±0.00	0.15±0.00	0.16±0.00	0.16±0.00
20:5n-3	*5*.*37*	2.52±0.04	2.60±0.07	2.51±0.05	2.60±0.13	2.74±0.06	2.62±0.05
22:2n-6	*0*.*08*	0.11±0.00	0.12±0.01	0.12±0.01	0.11±0.01	0.11±0.01	0.12±0.01
22:4n-6	*0*.*09*	0.07±0.00	0.07±0.00	0.08±0.01	0.08±0.00	0.08±0.00	0.07±0.00
22:5n-6	*0*.*22*	0.13±0.01	0.13±0.00	0.15±0.01	0.13±0.00	0.15±0.00	0.14±0.00
24:1n-9	*0*.*33*	0.23±0.01^a^	0.25±0.00^ab^	0.25±0.00^ab^	0.24±0.00^ab^	0.27±0.01^b^	0.26±0.01^ab^
22:5n-3	*1*.*62*	0.89±0.02	0.92±0.02	0.90±0.02	0.92±0.05	0.99±0.02	0.92±0.01
22:6n-3	*9*.*62*	5.43±0.23^a^	5.76±0.04^ab^	5.93±0.11^ab^	5.83±0.14^ab^	6.11±0.12^b^	5.87±0.03^ab^
24:5n-3	*0*.*2*	0.10±0.00	0.10±0.00	0.10±0.01	0.10±0.00	0.10±0.01	0.09±0.00
24:6n-3	*0*.*19*	0.15±0.00	0.17±0.01	0.17±0.01	0.16±0.01	0.16±0.01	0.17±0.02
SFA^2^	*27*.*47*	26.67±0.72	27.27±0.04	27.47±0.13	26.83±0.09	27.02±0.25	27.42±0.21
MUFA^2^	*42*.*28*	49.90±1.13	48.25±0.11	48.05±0.05	48.56±0.24	48.71±0.24	48.59±0.32
PUFA^2^	*30*.*25*	23.43±0.41	24.48±0.08	24.48±0.08	24.60±0.33	24.27±0.12	23.99±0.23
n-6 PUFA^2^	*10*.*14*	12.15±0.08^a^	12.59±0.07^b^	12.53±0.05^b^	12.67±0.04^b^	12.03±0.09^a^	12.14±0.17^a^
n-6 LC-PUFA^2^	*1*.*99*	2.17±0.06	2.27±0.03	2.31±0.02	2.28±0.02	2.26±0.01	2.25±0.01
n-3 PUFA^2^	*20*.*1*	11.28±0.34	11.89±0.13	11.94±0.10	11.93±0.33	12.24±0.18	11.85±0.07
n-3 LC-PUFA^2^	*17*.*69*	9.56±0.30^a^	10.06±0.12^ab^	10.13±0.09^ab^	10.13±0.31^ab^	10.61±0.18^b^	10.17±0.02^ab^
n-3/n-6 ratio^2^	*0*.*256*	0.123±0.007	0.117±0.006	0.126±0.004	0.118±0.007	0.129±0.009	0.121±0.008
Total Fatty acid(mg/g of lipid)	*775*.*1*	758.6±27.5	810.1±35.0	756.1±16.5	802.5±40.4	795.8±44.9	817.0±51.9

Initial fish not included in statistical analysis. Data are expressed as mean ± S.E.M., n = 3; N = 18. Values in the same row with different letters (a, b, c) are significantly different (P < 0.05; ANOVA and Student–Newman–Keuls post hoc test).

^1^ abbreviation as in Table 1.

^2^ abbreviation as in Table 2.

**Table 8:** The fatty acid composition (% of total fatty acids) and total fatty acid content (mg/g of lipid) of the fillets of Atlantic Salmon at commencement and the completion of the in vivo feeding trial.

		*Experimental treatments* ^*1*^					
*% of total fatty acids*	*Initial*	CD	LV	HV	LR	HR	VR
12:0	*0*.*08*	0.03±0.03	0.09±0.02	0.02±0.02	0.05±0.03	0.03±0.03	0.09±0.05
14:0	*3*.*69*	2.39±0.01	2.38±0.02	2.33±0.05	2.43±0.03	2.42±0.03	2.37±0.04
14:1n-5	*0*.*18*	0.16±0.00^a^	0.16±0.00^a^	0.16±0.00^a^	0.17±0.00^a^	0.19±0.00^b^	0.18±0.00^b^
16:0	*19*.*05*	19.65±0.08^b^	19.29±0.02^ab^	19.17±0.10^ab^	18.88±0.06^a^	18.93±0.15^a^	19.47±0.20^b^
16:1n-7	*6*.*65*	5.76±0.05^a^	5.76±0.03^a^	5.71±0.07^a^	5.87±0.06^a^	6.49±0.01^b^	6.39±0.04^b^
17:0	*0*.*36*	0.25±0.03	0.28±0.01	0.29±0.00	0.27±0.00	0.27±0.01	0.27±0.01
17:1n-7	*0*.*24*	0.18±0.00	0.19±0.02	0.19±0.01	0.18±0.01	0.26±0.07	0.20±0.00
18:0	*5*.*31*	5.70±0.07	5.63±0.01	5.64±0.05	5.43±0.04	5.43±0.09	5.59±0.07
18:1n-9	*26*.*55*	34.64±0.17	34.70±0.21	34.54±0.28	34.59±0.29	34.12±0.13	34.12±0.05
18:1n-7	*3*.*69*	3.56±0.01^a^	3.56±0.01^a^	3.57±0.01^a^	3.61±0.01^a^	3.81±0.02^c^	3.72±0.04^b^
18:2n-6	*7*.*48*	9.71±0.03^b^	9.84±0.08^b^	9.78±0.08^b^	9.76±0.07^b^	9.21±0.08^a^	9.18±0.01^a^
18:3n-6	*0*.*23*	0.30±0.01	0.32±0.00	0.31±0.00	0.30±0.01	0.30±0.01	0.31±0.01
18:3n-3	*1*.*15*	1.11±0.01^b^	1.13±0.01^b^	1.14±0.02^b^	1.13±0.01^b^	0.93±0.01^a^	0.93±0.01^a^
20:0	*0*.*23*	0.20±0.01	0.21±0.01	0.26±0.06	0.21±0.01	0.22±0.01	0.20±0.02
18:4n-3	*0*.*97*	0.55±0.02	0.58±0.00	0.58±0.00	0.56±0.00	0.55±0.00	0.56±0.00
20:1n-13	*0*.*32*	0.25±0.02	0.23±0.00	0.22±0.00	0.25±0.00	0.25±0.01	0.24±0.00
20:1n-11	*0*.*12*	0.10±0.01	0.09±0.00	0.09±0.00	0.09±0.00	0.10±0.01	0.10±0.00
20:1n-9	*1*.*13*	1.43±0.03	1.40±0.01	1.35±0.01	1.43±0.01	1.34±0.03	1.34±0.01
20:2n-6	*0*.*56*	0.95±0.12	0.84±0.02	0.81±0.02	0.86±0.02	0.77±0.03	0.77±0.02
20:3n-6	*0*.*31*	0.56±0.00	0.59±0.00	0.59±0.02	0.59±0.02	0.55±0.01	0.57±0.02
20:4n-6	*0*.*83*	0.70±0.02	0.70±0.01	0.72±0.03	0.75±0.02	0.76±0.02	0.75±0.01
20:3n-3	*0*.*1*	0.13±0.00^c^	0.12±0.00^abc^	0.12±0.00^bc^	0.12±0.00^bc^	0.11±0.00^ab^	0.10±0.00^a^
22:0	*0*.*12*	0.08±0.00	0.07±0.00	0.08±0.00	0.07±0.00	0.08±0.01	0.08±0.00
20:4n-3	*0*.*63*	0.41±0.00	0.42±0.01	0.43±0.00	0.41±0.02	0.42±0.01	0.42±0.03
22:1n-11	*0*.*28*	0.11±0.02	0.15±0.00	0.15±0.01	0.15±0.01	0.15±0.00	0.15±0.01
22:1n-9	*0*.*16*	0.14±0.00	0.15±0.00	0.16±0.01	0.15±0.01	0.15±0.00	0.15±0.01
20:5n-3	*5*.*06*	2.60±0.05^a^	2.67±0.02^ab^	2.74±0.04^ab^	2.74±0.06^ab^	2.86±0.08^b^	2.77±0.03^ab^
22:2n-6	*0*.*14*	0.19±0.06	0.19±0.03	0.18±0.02	0.18±0.01	0.21±0.01	0.19±0.00
22:4n-6	*0*.*09*	0.06±0.01	0.08±0.00	0.07±0.00	0.08±0.00	0.08±0.00	0.08±0.00
22:5n-6	*0*.*27*	0.14±0.00	0.15±0.00	0.15±0.01	0.16±0.01	0.16±0.01	0.16±0.01
24:1n-9	*0*.*3*	0.21±0.01	0.22±0.01	0.21±0.01	0.22±0.01	0.21±0.01	0.21±0.00
22:5n-3	*1*.*5*	0.85±0.02^a^	0.90±0.01^ab^	0.90±0.01^ab^	0.93±0.02^b^	0.98±0.01^c^	0.93±0.01^b^
22:6n-3	*12*.*04*	6.82±0.13	6.77±0.20	7.18±0.31	7.24±0.29	7.57±0.21	7.33±0.21
24:5n-3	*0*.*17*	0.06±0.01	0.07±0.00	0.07±0.00	0.08±0.01	0.08±0.01	0.07±0.00
24:6n-3	*0*.*03*	0.04±0.02	0.05±0.05	0.09±0.04	0.09±0.05	-	0.02±0.02
SFA^2^	*28*.*84*	28.31±0.13^b^	27.96±0.04^ab^	27.79±0.17^ab^	27.33±0.07^a^	27.39±0.20^a^	28.08±0.26^ab^
MUFA^2^	*39*.*6*	46.54±0.17	46.63±0.23	46.35±0.38	46.70±0.37	47.08±0.26	46.79±0.10
PUFA^2^	*31*.*55*	25.15±0.27	25.41±0.26	25.87±0.23	25.97±0.30	25.53±0.32	25.13±0.24
n-6 PUFA^2^	*9*.*91*	12.59±0.07^b^	12.71±0.06^b^	12.62±0.11^b^	12.67±0.03^b^	12.02±0.09^a^	12.00±0.05^a^
n-6 LC-PUFA^2^	*2*.*2*	2.59±0.07	2.55±0.05	2.53±0.05	2.61±0.04	2.52±0.02	2.52±0.04
n-3 PUFA^2^	*21*.*64*	12.56±0.20	12.70±0.26	13.24±0.30	13.29±0.32	13.51±0.29	13.13±0.19
n-3 LC-PUFA^2^	*19*.*53*	10.90±0.21	10.99±0.26	11.53±0.30	11.60±0.33	12.04±0.29	11.65±0.20
n-3/n-6 ratio^2^	*0*.*318*	0.13±0.001^a^	0.13±0.003^a^	0.13±0.004^a^	0.13±0.004^a^	0.15±0.004^b^	0.15±0.005^b^
Total Fatty Acid(mg/g lipid)	*690*.*7*	746.0±6.9	731.6±11.9	755.6±8.7	753.7±16.3	729.4±17.6	706.2±19.6

Initial fish not included in statistical analysis. Data are expressed as mean ± S.E.M., n = 3; N = 18. Values in the same row with different letters (a, b, c) are significantly different (P < 0.05; ANOVA and Student–Newman–Keuls post hoc test).

^1^ abbreviation as in Table 1.

^2^ abbreviation as in Table 2.

Amongst n-3 LC-PUFA, significant differences among dietary algal level were also observed. The fatty acid 20:3n-3 was significantly affected by diets, with lower levels recorded in the fillets of fish fed HR and VR, and lower levels recorded in the whole bodies of fish fed LR, compared to other treatments. The EPA (20:5n-3) and DPA (22:5n-3) content in fish fillets were significantly affected by diets, with the highest levels recorded in fish fed HR. DHA (22:6n-3) was on the other hand significantly higher in the whole body of fish fed HR, compared to all other treatments. This resulted in overall higher content of n-3 LC-PUFA in the whole bodies of fish fed HR. The variation in n-6 and n-3 fatty acid mentioned above, also resulted in significantly higher n-3/n-6 ratio in the fillets of fish fed HR and VR, compared to other treatments.

### Apparent in vivo fatty acid beta-oxidation and bioconversion

Statistically significant differences were observed for the apparent *in vivo* fatty acid β-oxidation for energy production among dietary groups (Table 9). Specifically, treatment HR showed relatively higher fatty acid β-oxidation rates for the majority of the SFA and MUFA compared with the other treatments, whereas in fish fed VR a significantly lower β-oxidation of alpha-linolenic acid (18:3n-3) was recorded. Only minor modifications of the apparent *in vivo* fatty acid bioconversion, as affected by dietary treatments, were recorded (Table 10). Specifically the elongation of 18:3n-3 to 20:3n-3 was up-regulated in fish fed the low inclusion of the two algal products (LV and LR) compared to the other treatments. No differences were recorded for the activities of any of the main enzymes (elongases, desaturases and peroxisomal chain shortening) for the ultimate production of DHA.

**Table 9:** The apparent in vivo fatty acid β-oxidation (nmol/g/day; deduced by the whole body fatty acid balance method) in Atlantic Salmon fed the experimental diets containing different fortification level of selected micronutrients.

	*Experimental treatments* ^*1*^ * *					* *
*FA (nmol/g/day)* ^*2*^	CD	LV	HV	LR	HR	VR
12:0	6.0±0.0^c^	4.7±0.1^c^	0.2±0.1^a^	5.2±0.3^c^	2.1±0.6^b^	1.5±0.6^b^
14:0	89.3±1.4^a^	71.7±7.4^a^	82.2±3.3^a^	74.1±7.7^a^	113.0±7.7^b^	75.6±12.4^a^
14:1n-5	12.4±0.8^b^	9.2±0.6^a^	9.4±0.2^a^	8.7±0.6^a^	12.8±0.7^b^	10.7±0.9^ab^
16:0	487.7±6.8^a^	380.0±43.7^a^	392.4±25.1^a^	396.2±50.3^a^	639.2±57.1^b^	411.9±68.3^a^
16:1n-7	181.4±3.0^ab^	148.9±16.4^a^	165.1±7.0^ab^	147.2±17.1^a^	223.5±17.9^b^	172.5±22.2^ab^
18:1n-7	31.7±2.4	17.4±7.8	25.0±1.9	16.4±8.4	36.1±8.4	17.5±9.4
18:0	79.1±3.2^ab^	47.5±10.4^a^	47.7±6.8^a^	61.7±14.3^a^	115.2±14.9^b^	51.0±19.1^a^
18:1n-9	501.0±135.6	514.1±83.7	592.2±35.7	506.6±96.0	728.9±95.4	454.1±111.0
20:0	1.8±1.1	2.5±0.3	2.5±0.0	3.5±0.3	4.3±0.9	3.2±0.8
20:1n-11	1.5±0.1^ab^	0.9±0.2^a^	1.7±0.1^ab^	0.9±0.2^a^	2.3±0.6^b^	1.8±0.3^ab^
22:1n-11	2.4±0.0	2.1±0.2	2.4±0.2	2.6±0.3	3.4±0.4	2.4±0.5
22:0	0.1±0.1	0.2±0.1	0.0±0.0	0.5±0.2	0.8±0.4	0.1±0.1
18:2n-6	182.9±17.2	137.1±26.5	165.3±11.5	129.9±27.7	185.9±29.0	108.1±34.1
20:4n-6	1.1±0.2	-^3^	0.4±0.2	0.5±0.5	2.9±1.5	0.8±0.8
22:4n-6	0.3±0.1	0.1±0.1	0.2±0.1	0.2±0.1	0.5±0.3	0.5±0.4
22:5n-6	0.1±0.1	0.0±0.0	0.1±0.1	0.0±0.0	0.2±0.1	0.1±0.1
18:3n-3	44.2±1.3^b^	37.0±2.9^b^	39.6±0.8^b^	36.4±2.9^b^	33.7±2.5^b^	23.9±5.1^a^
18:4n-3	20.0±0.1	15.9±2.3	18.2±0.8	18.2±1.6	22.3±2.0	16.3±2.5
20:4n-3	1.2±0.3	-	0.7±0.3	1.8±0.8	2.3±1.0	3.2±1.2
20:5n-3	99.3±1.8	65.9±16.0	86.5±6.1	71.3±18.1	105.6±17.8	57.2±25.3
*Total Fatty Acids*	1,743±173	1,455±217	1,631±98	1,482±242	2,235±257	1,412±309

(Only fatty acidrecording an apparent in vivo fatty acid β-oxidation are reported).

Data are expressed as mean ± S.E.M., n = 3; N = 18. Values in the same row with different letters (a, b, c) are significantly different (P < 0.05; ANOVA and Student–Newman–Keuls post hoc test).

^1^ abbreviation as in Table 1.

^2^ apparent *in vivo* fatty acid β-oxidation (nmol/g/day).

^3^ not detected.

**Table 10:** The apparent in vivo activity (nmol/g/day) of the key enzymes in fatty acid biosynthetic pathways (deduced by the whole body fatty acid balance method) in Atlantic Salmon fed the experimental diets.

	*Experimental treatments* ^*1*^			* *	* *	* *
*In vivo activity (nmol/g/day)* ^*2*^	CD	LV	HV	LR	HR	VR
*Fatty acid elongase*						
16:1n-7 to 18:1n-7	-^3^	-	-	1.0±1.0	-	-
18:0 to 20:0	0.1±0.1	-	-	-	-	-
18:1n-9 to 20:1n-9	25.9±0.4	31.6±2.9	24.6±1.1	29.5±5.8	18.3±4.6	30.0±5.4
20:1n-9 to 22:1n-9	3.1±0.0	4.7±0.5	4.0±0.4	3.8±1.0	2.8±1.0	4.9±0.9
22:1n-9 to 24:1n-9	1.3±0.0	2.5±0.3	2.1±0.2	2.0±0.6	1.5±0.6	2.9±0.5
20:0 to 22:0	1.5±0.9	-	0.1±0.1	-	-	0.1±0.1
18:2n-6 to 20:2n-6	25.0±0.5	28.1±1.6	24.8±0.2	26.5±2.9	21.5±1.8	26.0±2.0
20:2n-6 to 22:2n-6	3.5±0.2	3.9±0.5	3.6±0.3	3.7±0.7	3.3±0.2	3.7±0.2
18:3n-6 to 20:3n-6	12.7±0.1	16.6±2.2	14.1±1.9	14.8±2.5	14.4±1.3	14.5±2.4
20:4n-6 to 22:4n-6	-	0.3±0.3	0.7±0.7	0.2±0.2	0.3±0.3	0.1±0.1
22:4n-6 to24:4n-6	-	0.4±0.3	0.6±0.5	0.3±0.3	0.3±0.3	0.6±0.4
18:3n-3 to 20:3n-3	1.8±0.1^ab^	2.3±0.1^b^	1.8±0.1^ab^	2.4±0.3^b^	1.4±0.1^a^	2.0±0.2^ab^
18:4n-3 to 20:4n-3	-	0.9±0.5	-	-	-	-
20:5n-3 to 22:5n-3	41.1±0.5	64.4±12.0	47.3±6.1	61.1±14.4	47.4±11.1	66.7±19.0
22:5n-3 to 24:5n-3	42.8±1.2	64.0±10.8	50.5±6.3	61.5±13.1	49.3±9.1	67.0±16.8
*Fatty acid desaturase (Δ-6 and Δ-5)*						
18:2n-6 to 18:3n-6	14.0±0.2	19.8±2.9	16.7±2.5	17.8±3.4	16.0±1.9	17.7±3.6
24:4n-6 to 24:5n-6	0.0±0.0	0.4±0.3	0.6±0.5	0.3±0.3	0.3±0.3	0.6±0.4
24:5n-3 to 24:6n-3	41.1±1.2	62.1±10.6	49.0±6.3	59.6±12.8	47.5±9.1	65.3±16.4
20:3n-6 to 20:4n-6	-	1.7±1.4	1.1±1.1	1.4±1.0	0.3±0.3	1.6±1.0
*Peroxisomal chain shortening*						
24:5n-6 to 22:5n-6	-	0.4±0.3	0.6±0.5	0.3±0.3	0.3±0.3	0.6±0.4
24:6n-3 to 22:6n-3	37.2±1.5	57.4±10.2	45.0±5.9	55.3±12.3	43.6±8.8	60.6±15.4

Data are expressed as mean ± S.E.M., n = 3; N = 18. Values in the same row with different letters (a, b) are significantly different (P < 0.05; ANOVA and Student–Newman–Keuls post hoc test).

^1^ abbreviation as in Table 1.

^2^ apparent *in vivo* activity (nmol/g/day).

^3^ not detected.

## Discussion

The results from the present study indicate that inclusion of algae product Verdemin and Rosamin at levels of 2.5 and 5.0% in practical diets did not cause any significant effect on growth performance, fish biometry macronutrient digestibility and muscle fatty acid composition of Atlantic Salmon, with the exception of feed intake and FCR for HR treatment, and a slight reduction in protein digestibility for both tested products. Accordingly, previous studies testing the inclusion of 5% of *Ulva* spp. meal in feed for carnivorous fish like European sea bass (*Dicentrarchus labrax)* [16] and rainbow trout [43] reported no effect on growth performance. Nevertheless, other studies that tested dietary inclusion up to 10–15% of algae meal in feed for fishes with more amylase activity (e.g. herbivorous and/or omnivorous species) [44] which are reported to be able to digest algal products more efficiently [10,45], such as Nile tilapia [46,47], common carp (*Cyprinus carpio*) [15] gilthead sea bream (*Sparus aurata*) [22], reported significant improvement in growth performances, feed efficiency, nutrient utilisation and body composition. Accordingly, the response of fish to dietary algal inclusion is well documented to be species-specific. In the present study a significant negative effect of both algal product inclusion on protein digestibility was observed. It has been suggested that carnivorous fish, like Atlantic Salmon, are not particularly efficient in digesting particle nutrients from algae. Accordingly, it may be suggested that the observed negative effect on protein digestibility in diets formulated with the inclusion of algal products was resulting from a lower digestibility of the actual protein contained in the algal products. However, previous studies suggest that fish are not able to digest more than 45–56% protein from algae [45], not because of protein quality, but because of the limited ability in the hydrolysis of complex polysaccharides present in algae. Therefore the negative effect recorded in this study on nutrient digestibility was likely not due to an actual lower digestibility of those nutrients, but was due to a negative effect of the complex polysaccharides contained in algal products. Additionally, it is also important to considerer that in the present study the total protein content originating from the algae inclusion was only 1–2% (low to high inclusion) of the total protein content of the diet, and therefore it is more likely that the recorded negative effect on protein digestibility is due to a negative interaction between some components of the algal products and proteolytic enzymes.

General beneficial effects of the use of algae in finfish nutrition are not commonly reflected in a direct positive effect on growth, but more typically reflected in effects of some specific physiological activities [21]. For example, positive effects of algal products in fish feed have been reported on improved liver functions, stress response and starvation tolerance [43], and in overall increased carcass quality and health status, and in particular improved health of the intestinal mucosa [11,21,23]. Admittedly, the present study was not designed to address these specific scientific questions and more compressive studies are warrant, specifically towards assessing the potential beneficial effects of the inclusion of algae in aquafeed with respect to fish welfare and stress tolerance, which is an increasingly important issue of the current aquaculture industry.

The present study also found an increase in the n-3 LC-PUFA content in whole body of fish fed the algal products. Accordingly, similar results have been reported previously in studies in Atlantic salmon fed with a commercial blend of seaweed [48] and in mice feed with the algal carotenoid fucoxanthin and fucoxanthinol, where a remarkable increase in the polyunsaturated fatty acid content of the liver was observed [30,34,49]. All this evidence, clearly displays the importance of these bioactive carotenoids found in algae. Importantly, the observed increased in n-3 LC-PUFA content of fish tissues, though statistically significant, from a nutritional point of view for consumers can be considered as minimal. Accordingly, it can be reported that it is unlikely that the improved n-3 LC-PUFA content of fish tissues resulting from dietary algal product inclusion could compensate for the possible reduction of n-3 LC-PUFA resulting from a reduction of dietary fish oil inclusion.

This study showed that Verdemin and Rosamin seem to have limited potential for their inclusion in salmon feed, with respect simply to overall performances. However, some interesting biologic activities were observed, that could be of interest for specific applications, and warrant further specifically designed studies.
